# Supplementary material for: Pyruvate Dehydrogenase Kinase 1 inhibition mediated oxidative phosphorylation enhancement in cartilage promotes osteoarthritis progression
Source: BMC Musculoskelet Disord. 2023 Jul 20;24:597. doi: 10.1186/s12891-023-06585-6 (PMC10357736; doi:10.1186/s12891-023-06585-6)
Supplement: Supplementary file 1 — Additional file 1. [file 12891_2023_6585_MOESM1_ESM.zip › BP.Gsea.1653623667859/GOBP_EMBRYONIC_HEMOPOIESIS.html]

Details for gene set GOBP\_EMBRYONIC\_HEMOPOIESIS[GSEA]

|  || Dataset | 55457\_collapsed\_to\_symbols.cls.cls #OA\_versus\_normal.cls.cls #OA\_versus\_normal\_repos |
| Phenotype | cls.cls#OA\_versus\_normal\_repos |
| Upregulated in class | normal |
| GeneSet | GOBP\_EMBRYONIC\_HEMOPOIESIS |
| Enrichment Score (ES) | -0.5556515 |
| Normalized Enrichment Score (NES) | -1.7591114 |
| Nominal p-value | 0.0036630037 |
| FDR q-value | 0.110649936 |
| FWER p-Value | 0.998 |
Table: GSEA Results Summary

  

Fig 1: Enrichment plot: GOBP\_EMBRYONIC\_HEMOPOIESIS      
 Profile of the Running ES Score & Positions of GeneSet Members on the Rank Ordered List

  

| SYMBOL | TITLE | RANK IN GENE LIST | RANK METRIC SCORE | RUNNING ES | CORE ENRICHMENT || 1 | FLT3LG | fms related receptor tyrosine kinase 3 ligand [Source:HGNC Symbol;Acc:HGNC:3766] | 894 | 0.544 | -0.0007 | No |
| 2 | KIT | "KIT proto-oncogene, receptor tyrosine kinase [Source:HGNC Symbol;Acc:HGNC:6342]" | 2117 | 0.348 | -0.0517 | No |
| 3 | PBX1 | PBX homeobox 1 [Source:HGNC Symbol;Acc:HGNC:8632] | 3887 | 0.166 | -0.1681 | No |
| 4 | SH2B3 | SH2B adaptor protein 3 [Source:HGNC Symbol;Acc:HGNC:29605] | 4960 | 0.076 | -0.2418 | No |
| 5 | TAL1 | "TAL bHLH transcription factor 1, erythroid differentiation factor [Source:HGNC Symbol;Acc:HGNC:11556]" | 5406 | 0.040 | -0.2714 | No |
| 6 | CCDC134 | coiled-coil domain containing 134 [Source:HGNC Symbol;Acc:HGNC:26185] | 6436 | -0.038 | -0.3465 | No |
| 7 | TPO | thyroid peroxidase [Source:HGNC Symbol;Acc:HGNC:12015] | 6467 | -0.040 | -0.3438 | No |
| 8 | TGFBR2 | transforming growth factor beta receptor 2 [Source:HGNC Symbol;Acc:HGNC:11773] | 8241 | -0.174 | -0.4595 | No |
| 9 | THOC5 | THO complex 5 [Source:HGNC Symbol;Acc:HGNC:19074] | 8549 | -0.198 | -0.4584 | No |
| 10 | KMT2A | lysine methyltransferase 2A [Source:HGNC Symbol;Acc:HGNC:7132] | 8807 | -0.217 | -0.4509 | No |
| 11 | IL3 | interleukin 3 [Source:HGNC Symbol;Acc:HGNC:6011] | 9850 | -0.306 | -0.4931 | No |
| 12 | STK3 | serine/threonine kinase 3 [Source:HGNC Symbol;Acc:HGNC:11406] | 10656 | -0.388 | -0.5066 | Yes |
| 13 | GATA1 | GATA binding protein 1 [Source:HGNC Symbol;Acc:HGNC:4170] | 10958 | -0.425 | -0.4763 | Yes |
| 14 | GATA2 | GATA binding protein 2 [Source:HGNC Symbol;Acc:HGNC:4171] | 11058 | -0.439 | -0.4286 | Yes |
| 15 | STK4 | serine/threonine kinase 4 [Source:HGNC Symbol;Acc:HGNC:11408] | 11074 | -0.441 | -0.3740 | Yes |
| 16 | MED1 | mediator complex subunit 1 [Source:HGNC Symbol;Acc:HGNC:9234] | 11146 | -0.450 | -0.3228 | Yes |
| 17 | KDR | kinase insert domain receptor [Source:HGNC Symbol;Acc:HGNC:6307] | 11454 | -0.496 | -0.2839 | Yes |
| 18 | KITLG | KIT ligand [Source:HGNC Symbol;Acc:HGNC:6343] | 11516 | -0.503 | -0.2251 | Yes |
| 19 | HIF1A | hypoxia inducible factor 1 subunit alpha [Source:HGNC Symbol;Acc:HGNC:4910] | 11833 | -0.556 | -0.1795 | Yes |
| 20 | ATF4 | activating transcription factor 4 [Source:HGNC Symbol;Acc:HGNC:786] | 11999 | -0.593 | -0.1174 | Yes |
| 21 | GATA3 | GATA binding protein 3 [Source:HGNC Symbol;Acc:HGNC:4172] | 12276 | -0.670 | -0.0543 | Yes |
| 22 | VEGFA | vascular endothelial growth factor A [Source:HGNC Symbol;Acc:HGNC:12680] | 12629 | -0.813 | 0.0210 | Yes |
Table: GSEA details [plain text format]

  

Fig 2: GOBP\_EMBRYONIC\_HEMOPOIESIS      
 Blue-Pink O' Gram in the Space of the Analyzed GeneSet

  

Fig 3: GOBP\_EMBRYONIC\_HEMOPOIESIS: Random ES distribution      
 Gene set null distribution of ES for **GOBP\_EMBRYONIC\_HEMOPOIESIS**
